# Supplementary material for: ABC model for cost estimation of custom implants by Additive Manufacturing
Source: PLoS One. 2024 May 29;19(5):e0301440. doi: 10.1371/journal.pone.0301440 (PMC11135763; doi:10.1371/journal.pone.0301440)

Appendix 1

Work zone Report

finger3

Description

Job Execution History

- [x] Last execution only
- [ ] Work Completed
- [ ] User Stop Request
- [ ] User Skip Request
- [ ] Last Active Slice Reached
- [ ] Splitting Part Mixed [illegible]
- [ ] Unknown [illegible] Supply Chamber Security Quote
- [ ] Reached Work Chamber Security Quote
- [ ] Insufficient Gas Pressure
- [ ] Power Failure
- [ ] Laser Failure
- [ ] Exceeded T/H Thresholds
- [ ] Unexpected Error
- [ ] Motor Error
- [ ] Gas Regulation Alarm
- [ ] Air Velocity Regulation Alarm
- [ ] Unacceptable Gas Level
- [ ] Unacceptable Air Velocity
- [ ] Filter Exhaust
- [ ] Laser Scanner Failure
- [ ] Unable To Lock Door
- [ ] EtherCAT Master Alarm
- [ ]
- [ ]

	Begin	End
**Date/Time:**	2023-Jun-07 10:43:16	2023-Jun-07 12:15:17
**Job duration:**	1 hours, 32 minutes, 0 seconds	
**Slice:**	1	1033
**Supply chamber quote (mm):**	46.186 [user -24.918]	89.066 [user -67.797]
**Work chamber quote (mm):**	86.258 [user 36.743]	65.598 [user 57.402]
**Average Gas Regulation (l/min):**	0.892	
**Total Gas Consumption (l):**	475.845	
**Filter Life (%):**	7.4	14.9
**Stop Reason:**	Work Completed	

Material Parameters

Material profile name:last used -316L-A

Laser Power (W):	100
Laser speed (mm/sec):	600
Chambers Retraction (mm):	0
Coater Work Speed (mm/sec):	80
Coater Return Speed (mm/sec):	250
Coater Pre-Coating Speed (mm/sec):	160
Use Dynamic Over Supply:	false
Over Supply Factor (%):	200
Minimun Over Supply Factor (%):	110
Fan speed (m/sec):	3
Oxygen level (%):	0.3
Laser Focus Shift (mm):	0
Enforce CAM parameters:	true
Wobbel Amplitude (mm):	0
Wobbel Frequency (Hz):	0
Enable Wobbel Function:	false
Inert Gas Type:	Nitrogen
First Layer Iterations:	1
Scanner Behavior:	MixedAdvancedSkywriting
Process Camera Acquisition Enabled:	false
Minimum Time Between Exposure:	0
Increase Supply Factor for First Slices:	true
Number Slice with Increased Over Supply:	40
Value Over Supply First Slices (%):	400
Powder Density (kg/dm^3):	0
Substrate Type:	3
Grid Size (mm):	2.5
Grid Angle (°):	0
Grid Margin (mm):	5
Grid Laser Beam Speed (mm/sec):	500
Grid Laser Power (W):	100
[OverwritePulsingParams]:	false
[In_Skin_Pulsing]:	false 0 0 false 0 0 false 0 0
[Up_Skin_Pulsing]:	false 0 0 false 0 0 false 0 0
[Down_Skin_Pulsing]:	false 0 0 false 0 0 false 0 0
[Core_Skin_Pulsing]:	false 0 0 false 0 0 false 0 0
[Support_Pulsing]:	false 0 0 false 0 0 false 0 0

Original geometry data file information

Index:	1
Original geometry data file:	E:\Finger\Finger.WZA
Original geometry part name:	Finger
Slicing:	0 layers (height: 0.020 mm)
Offset (X, Y):	0.000, 0.000
Bounding box (X, Y, Width, Height):	-6.459, -5.508, 7.614, 8.966
Laser Parameters:	*See detailed CAM parameters report.*

finger3

General:

Builjob Name:	finger3
Job Id:	472
Workzone Name:	finger3
Production Date:	07/06/2023
Start Buildjob:	07/06/2023 10:15:27
End Buildjob:	07/06/2023 12:15:17
Runtime:	1h 59m
Layers:	1033/1033
Build height:	20.66 mm
Reason For Job End:	WorkCompleted
Operator:	

Material:

TYPE:

LOT Nr.:

Order Information:

Order Number:

Job Number:

Notes:

Machine Information:

Serial Number:	LS0010382
Hmi Software Version:	

Condition Monitoring:

Ambient humidity

Status: Ok
Status message: Everything Ok
Min: 55.20 % r.H.
Max: 69.80 % r.H.
Mean: 65.31 % r.H.

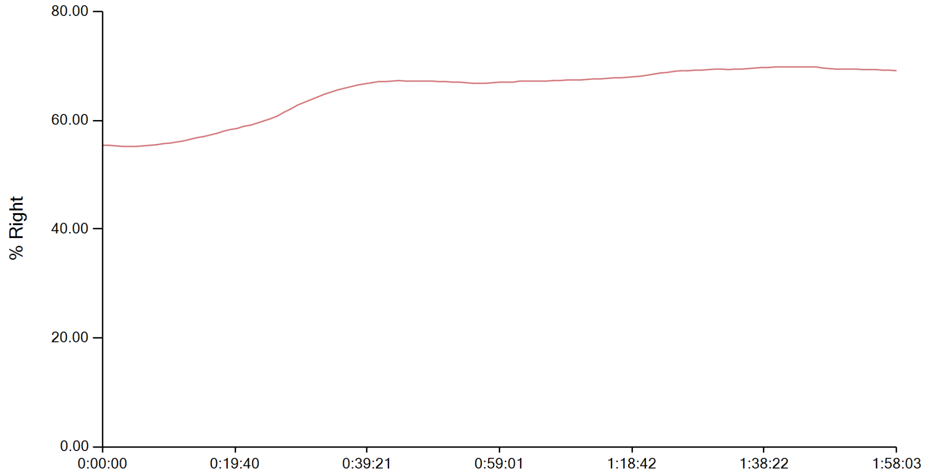

Ambient temperature

Status: Ok
Status message: Everything Ok
Min: 21.80 °C
Max: 23.10 °C
Mean: 22.45 °C

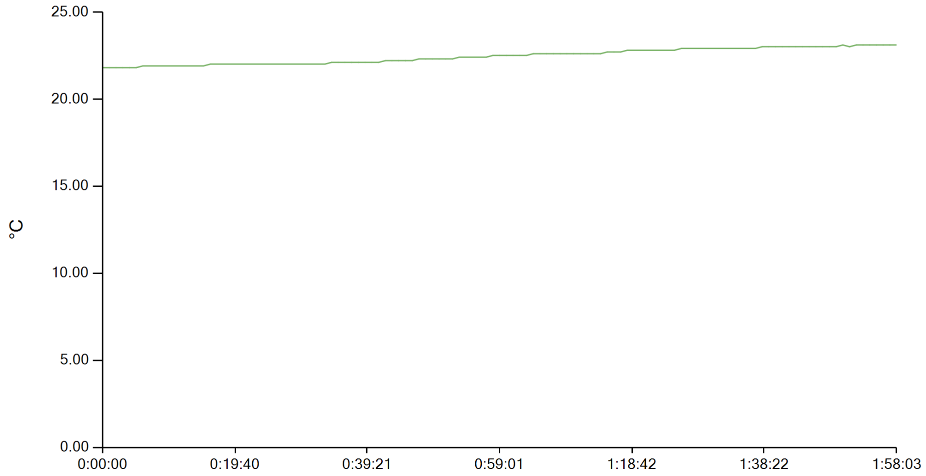

Filter Status

Status: Ok
Status message: Everything Ok
Min: 0.00 %
Max: 14.90 %
Mean: 11.29 %

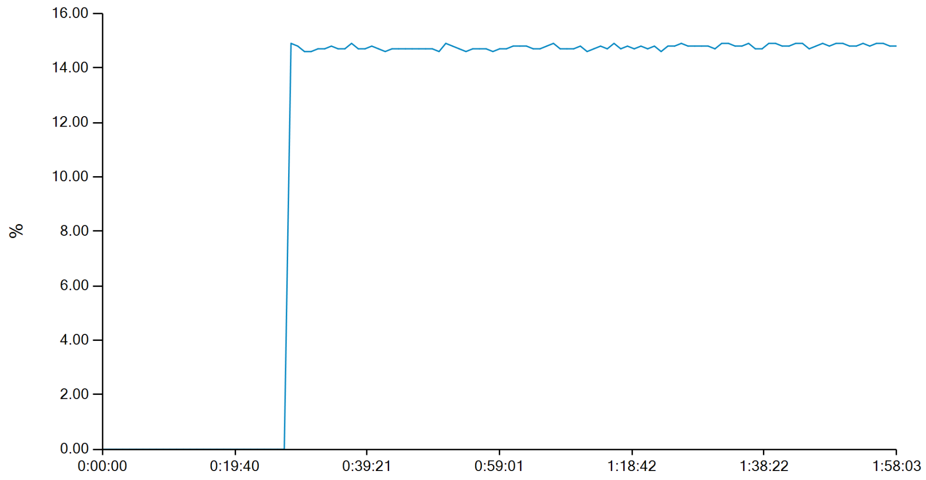

Inert gas flow

Status: Ok
Status message: Everything Ok
Min: 0.00 l/min
Max: 50.00 l/min
Mean: 4.03 l/min

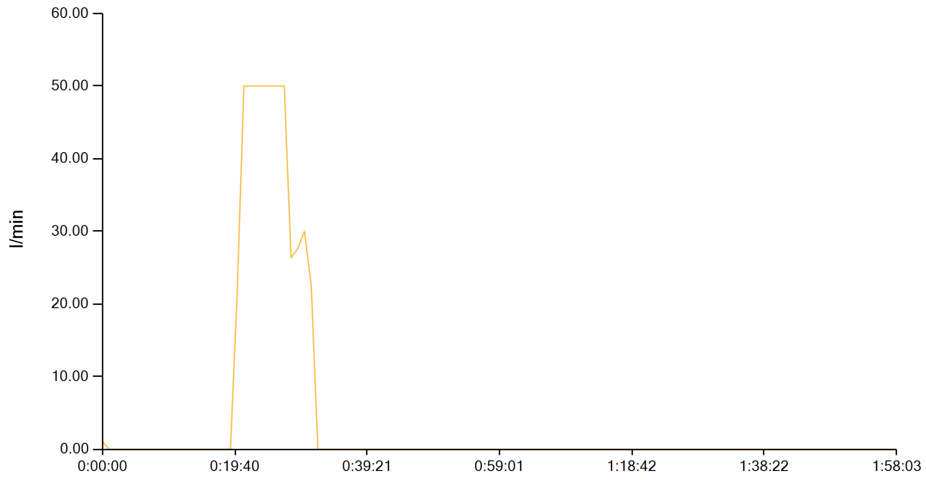

O2 Content (Process)

Status: Ok
Status message: Everything Ok
Min: 0.02 %
Max: 20.05 %
Mean: 3.59 %
Setpoint: 0.3% ±5%

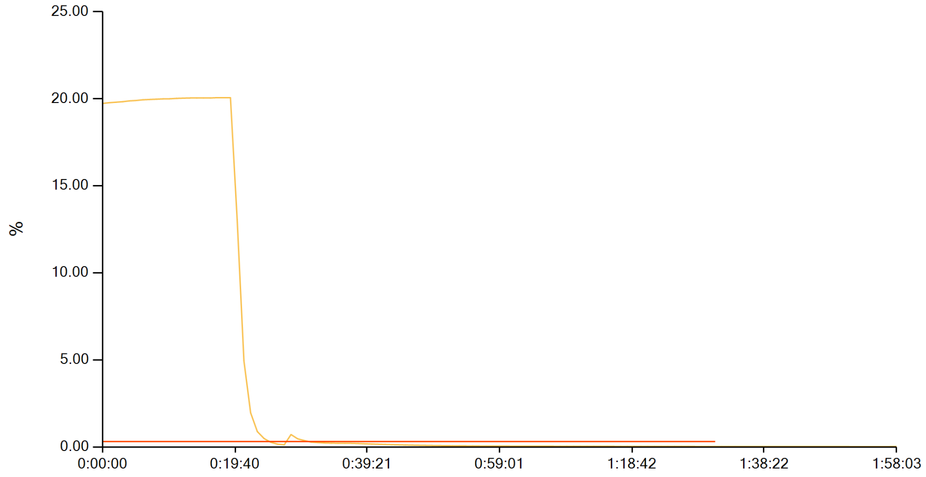

Shielding gas flow rate

Status: Ok
Status message: Everything Ok
Min: 0.07 m/s
Max: 3.02 m/s
Mean: 2.33 m/s

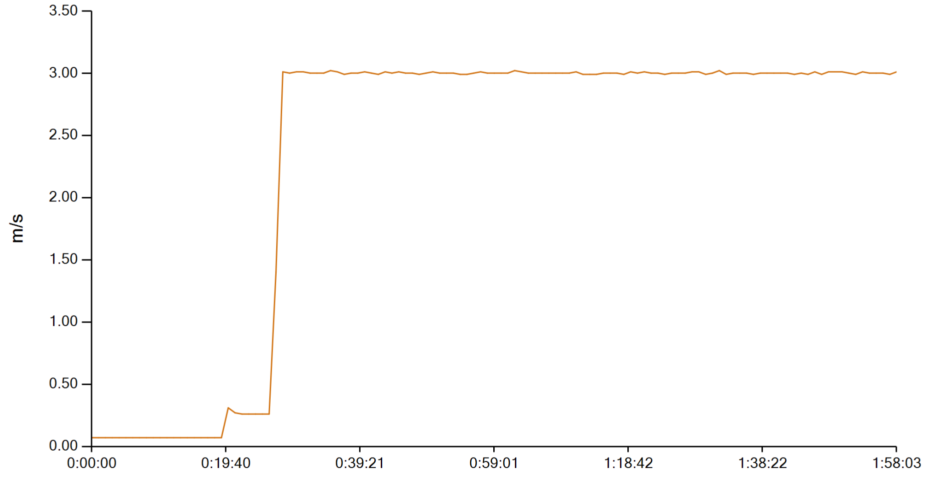

Performance Monitoring:

Used powder volume:	0.34 l
Used gas volume for inertization:	407.92 l
Inertization duration:	29 min
Used gas volume during build:	80.40 l
Total Coating time for build job:	58 min
Total Melting time for build job:	13 min

Details Display Screen in the Machine

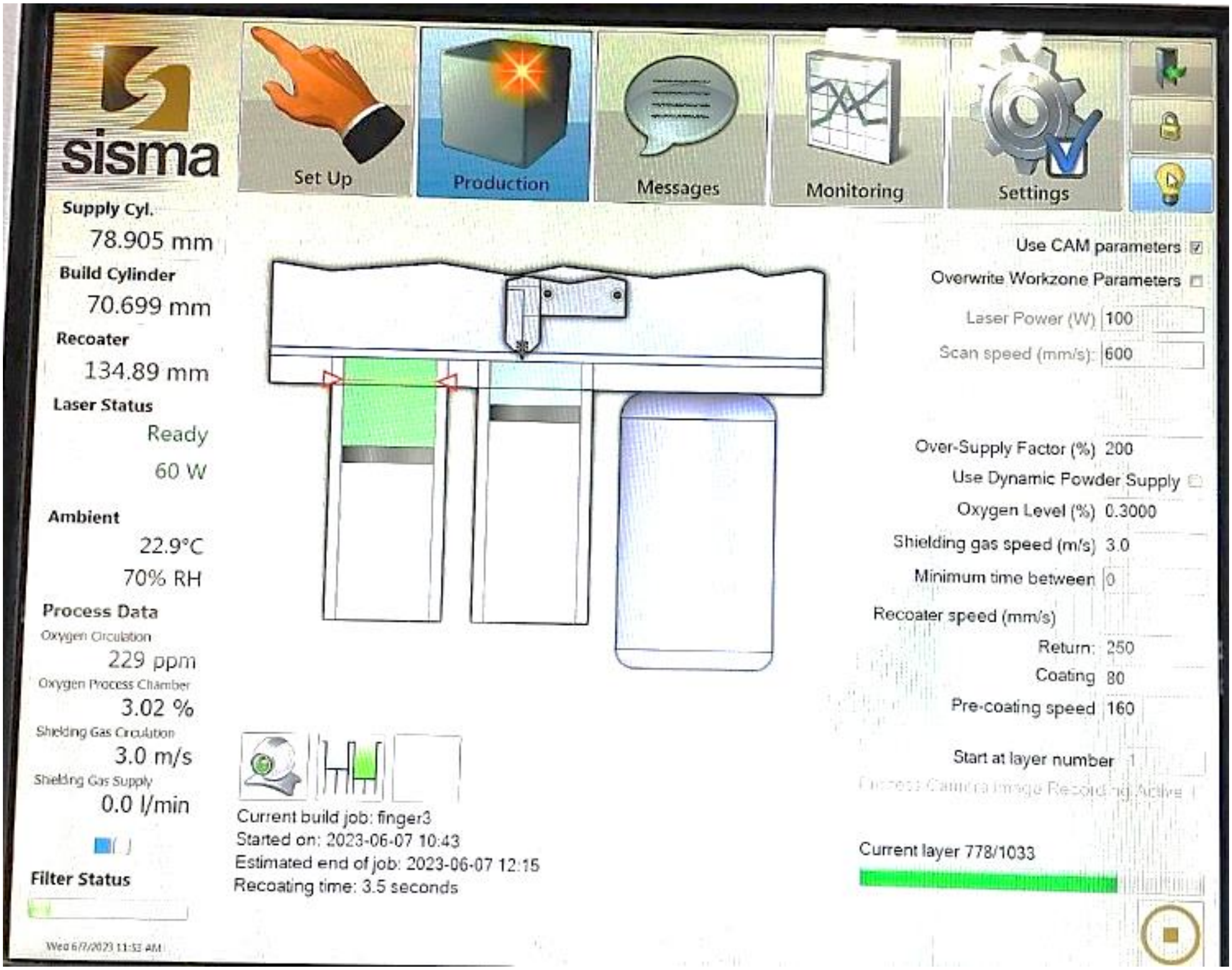

Supplement: S1 Appendix — (PDF) [file pone.0301440.s001.pdf]
