# Supplementary material for: ABC model for cost estimation of custom implants by Additive Manufacturing
Source: PLoS One. 2024 May 29;19(5):e0301440. doi: 10.1371/journal.pone.0301440 (PMC11135763; doi:10.1371/journal.pone.0301440)
Supplement: Attachment — Submitted filename: PONE D 23 42855 Details.docx [file pone.0301440.s002.docx]

Reviewer’s comments

The content of the paper is practical and has more use to many stake-holders such as patients, manufacturers, doctors and salespersons.

Thus, calculations should be more clear and add table of comparison allowing for better and easy selection of items.

1. Grammatical mistakes are to be corrected
a. Yang and Li had [5] developed – should be in present simple tense
b. Ulu et al. (2019) [6] suggested - should be in present simple tense
2. Referencing should be corrected, in some places only the [number] but in some cases both names and number as in the example.
a. Yang and Li had [5]
b. Ulu et al. (2019) [6]
3. The total cost (PT) should be corrected as 𝑃_𝑇_
4. The term used in research for ‘Shortened form’ is Abbreviation.
5. Move “Table 1: Pre-processing, Processing, and Post-processing Cost Estimation” before the calculations as placed as the Table 2.
6. PETG has not been defined.
7. Formatting errors were notified in few places.
8. Mistakes in the List of References has to be corrected;
   a. Name of the journal missing: [16], [17]
   b. et al.
9. Start from a Capital letter: [2]
