# Supplementary material for: ABC model for cost estimation of custom implants by Additive Manufacturing
Source: PLoS One. 2024 May 29;19(5):e0301440. doi: 10.1371/journal.pone.0301440 (PMC11135763; doi:10.1371/journal.pone.0301440)
Supplement: Attachment — Submitted filename: Plos one Review 2 PONE D 23 42855 Details.docx [file pone.0301440.s004.docx]

Reviewer’s comments on 11 03 2024 “ABC Model for Cost Estimation of Custom Implants by Additive Manufacturing”

Please refer further comments.

1. The novelty of this article which has been highlighted, do not prominent in the title, the abstract or in keywords. So re-write title, the abstract and keywords, removing unwanted definitions and clarifications.

- Rather writing ‘custom implants’ in the title, the authors can replace by customizing medical implants in the title and better little change the abstract and keywords accordingly. There, general or unnecessary explanations in the abstract can be removed.
- Nowadays, Additive Manufacturing (AM), often known as 3D printing, is a technology that is making significant progress in customizing medical implants for individuals.
- According to the authors, the novelties of this study enables the creation of complex geometries impossible with traditional production methods by implanting for unusually shaped areas, such as the skull or pelvis, can benefit greatly from this innovation.
- we are utilizing the MYSINT 100 for manufacturing custom implants, offering advantages such as high precision, design flexibility and efficient production. Its selective laser melting technology allows for intricate and patient-specific implant designs, contributing to better fit and enhanced functionality. The MYSINT 100's capabilities in processing various materials make it suitable for a range of implant applications, providing a reliable and customizable solution in AM for medical purposes.
- The authors aim to represent a common and diverse set of materials used in AM and to make the research more realistic. As, plastic would no longer be useful for real-world application but would serve merely as an experimental part thus limited to use only metal, as it is medically and practically suitable for the patient

2. in the Abstract

Computer-aided design (CAD) model – Change aided design to capital letters and write as Computer-Aided Design

3. Formatting errors have to be removed.

• Overhead Cost (justify the paragraph,

The overhead costs during the processing phase, administrative services, lighting, etc. are proportioned based on the setup and build time (𝑇𝑏𝑠). This rate (𝐻2) could deviate from preprocessing, as environments during these operations vary, hence, the overhead cost (𝐶𝑂𝐻2 ) is calculated as:

This ensures that overhead costs are accurately associated with the processing phase.

c. Post-processing Activity (check Tabs, font size and justification of the paragraph)

Post-processing in AM entails finishing and inspection, including steps like heat treating. Post-processing costs cover material, machinery, labor, energy, inspection, and overhead. The formula for the postprocessing cost is:

4. future work

In addition to incorporating visual aids such as charts or graphs to represent the cost breakdown more intuitively to make visualizations enhance the reader's understanding and facilitate a quicker grasp of complex information, you may develop a software in the future studies.

……………………………………………………………………………………………………………………………………..

Reviewer’s comments 01

The content of the paper is practical and has more use to many stake-holders such as patients, manufacturers, doctors and salespersons.

Thus, calculations should be more clear and add table of comparison allowing for better and easy selection of items.

1. Grammatical mistakes are to be corrected
a. Yang and Li had [5] developed – should be in present simple tense
b. Ulu et al. (2019) [6] suggested - should be in present simple tense
2. Referencing should be corrected, in some places only the [number] but in some cases both names and number as in the example.
a. Yang and Li had [5]
b. Ulu et al. (2019) [6]
3. The total cost (PT) should be corrected as 𝑃_𝑇_
4. The term used in research for ‘Shortened form’ is Abbreviation.
5. Move “Table 1: Pre-processing, Processing, and Post-processing Cost Estimation” before the calculations as placed as the Table 2.
6. PETG has not been defined.
7. Formatting errors were notified in few places.
8. Mistakes in the List of References has to be corrected;
   a. Name of the journal missing: [16], [17]
   b. et al.
9. Start from a Capital letter: [2]
